# Functional CRISPR‐Cas9 knockout screening of the genetic determinants of human fibroblast migration propensity

**DOI:** 10.1002/btpr.70076

**Published:** 2025-10-02

**Authors:** Antonio Mazzei, Sebastian Martewicz, Ramin Amiri, Meihua Cui, Nicola Elvassore, Camilla Luni

**Affiliations:** ^1^ Department of Civil, Chemical, Environmental and Materials Engineering (DICAM) University of Bologna Bologna Italy; ^2^ Shanghai Institute for Advanced Immunochemical Studies (SIAIS) ShanghaiTech University Shanghai China; ^3^ Department of Industrial Engineering University of Padova Padova Italy; ^4^ Venetian Institute of Molecular Medicine Padova Italy

**Keywords:** Crispr, functional screening, genetic screening, GPCR, migration, olfactory receptor

## Abstract

Directional cell migration plays a central role in a wide range of physiological and pathological conditions, such as embryonic development or tumor metastasis. Steps involved in cell migration include cell polarization, formation of membrane protrusions at the cell front side and adhesion disassembly at the rear side, and a general cytoskeletal rearrangement. Overall, it is a complex phenomenon at the interface between mechanical forces and biochemical signaling, with cell‐specific and context‐specific molecular events acting in the process. Here, we focus on human fibroblast migration induced by a biochemical gradient with an approach that connects the identification of molecular players with the actual mechanical function. We show how to screen for genes and miRNAs involved in migration by the direct integration of a high‐throughput gene editing method, the CRISPR‐Cas9 knockout pool screening, and a well‐established functional assay, the transwell migration assay. Moreover, the screening has been performed after an expansion step aiming at the removal of all the essential genes and miRNAs, so as to identify targets related to the cell migratory ability without affecting other major cellular functions. The results confirm known genes involved in migration, but also highlight new candidates. This work establishes a methodological advancement in the use of CRISPR technology for functional screening and represents a resource for candidate genes and miRNAs playing a role in human fibroblast directional migration under biochemical gradient.

## 1 INTRODUCTION

Directional cell migration plays a central role in a wide range of physiological and pathological processes, including embryogenesis, tissue homeostasis, wound healing, immune response, chronic inflammation, and cancer.^1^ It is initiated by extracellular cues such as biochemical or mechanical force gradients.^2^ Despite different types of cells, such as fibroblasts, keratocytes, and leukocytes, being able to migrate, there is a continuum of migration modes that involve cell polarization, formation of membrane protrusions at the cell front side, and adhesion disassembly at the rear side, with traction forces involved in cytoskeletal rearrangements.^3^ Within this general framework, different cells in different environments may show specificities at the molecular level, for example, expressing various cell surface adhesion receptors.^2^ Thus, the ability to modulate the regulatory mechanisms underlying migration in specific pathological contexts requires the identification of precise cell‐specific pharmacological targets. Technological advancements for high‐throughput screening can be a powerful tool in this direction.

Clustered regularly interspaced short palindromic repeats (CRISPR)‐Cas technology development has been a breakthrough in genomic engineering.^4^ This editing tool is essentially based on the complementary action of two parts: a single guide RNA (sgRNA), which is a specific RNA sequence that recognizes the target DNA region of interest, and a Cas nuclease, which is directed by the sgRNA to that DNA region for editing. In pooled CRISPR‐Cas knockout screens, a library of sgRNAs is transduced in bulk into the cell population, with individual cells statistically receiving at most one sgRNA. Gene‐edited cells are then challenged with a selective pressure, and finally, sgRNAs are counted to detect their depletion or enrichment. Thus, pooled CRISPR‐Cas screening is an important tool for genome‐wide hypothesis generation. Currently, most studies have been performed using viability or altered proliferation in the presence of specific environmental perturbations as the selective pressure that alters the balance between the different sgRNAs in the population.^5^ More recently, to extend the phenotypic characterization of pooled perturbations, gene editing has been coupled with single‐cell sequencing,^6^, ^7^, ^8^, ^9^ assay for transposase‐accessible chromatin using sequencing (ATAC‐seq),^10^ protein detection by mass cytometry,^11^ and optical phenotypic readout coupled with sequencing of sgRNAs with spatial resolution.^12^, ^13^, ^14^

Here, we focused on the identification of the genetic determinants of human fibroblast migration propensity in the presence of a biochemical gradient. We use CRISPR‐Cas9 knockout screening directly integrated with a functional assay for investigating cell migration. Specifically, we used the transwell migration assay, a standard in vitro method to investigate the chemotaxis of cells through a porous membrane, after the establishment of a biochemical gradient produced by two medium‐filled compartments of different composition. This assay assesses the spontaneous ability of the cells to actively migrate in three‐dimensional (3D) environments through the narrow constrictions of the porous membrane.

## 2 MATERIALS AND METHODS

### 2.1 Cell Cultures

Human Foreskin Fibroblasts (HFF‐1, SCRC‐1041, ATCC) were maintained in Dulbecco's modified Eagle Medium (DMEM, cat. 11965, ThermoScientific) supplemented with 15% fetal bovine serum (FBS, ThermoScientific) at 37°C and 5% CO_2_. Cells were maintained on culture‐ware (including transwells, see below) coated with Matrigel growth factor reduced (MGFR, 50 μg/mL overnight at 4°C and 1 h at 37°C, cat. 354230, Corning) and passaged by 0.25% trypsin digestion (ThermoScientific) before reaching confluency. Stocks of cells frozen in 90% FBS + 10% DMSO (Sigma‐Aldrich) were thawed in complete medium and tested for mycoplasma contamination.

### 2.2 Preliminary transwell experiment

Cells were seeded at 500 cell/mm^2^ on the top part of transwells (33 mm Transwell® with 3.0‐μm or 5.0‐μm pore polycarbonate membrane insert in 24‐well multiwell plates, Corning) pre‐coated with MGFR. The top chamber contained media at 1% FBS and the bottom chamber at 15% FBS to generate a biochemical gradient. After 48 h, the membranes were fixed with paraformaldehyde (PFA) 4% (10 min at room temperature) and stained with Hoechst33342 for nuclei detection. Cells were fixed and cut from the plastic supports to allow confocal imaging of both sides of the insert (Zeiss LSM710 laser‐scanning confocal microscope).

### 2.3 sgRNA library amplification and lentivirus production

Human Genome‐Scale CRISPR Knock‐Out (GeCKO) lentiviral pooled library (GeCKO v2, library A, cat. 1,000,000,048, Addgene) was a gift from Feng Zhang,^15^ and contains 3 sgRNAs per gene, 4 sgRNAs per miRNA, and 1000 control sgRNAs, *Streptococcus pyogenes* Cas9 nuclease within the lentiviral backbone, and puromycin selectable marker. Library amplification and lentivirus production were performed as previously described.^16^

### 2.4 Crispr‐Cas9 knock‐out screening during transwell migration assay

Cells were thawed at p20 and seeded in flask at 100 cell/mm^2^. After 3 days, cells were replated at 100 cell/mm^2^ and further expanded. After 4 more days, about 70 × 10^6^ cells were seeded at 200 cell/mm^2^ in 4 T875 five‐layer flasks. Six hours after seeding, medium was changed to medium supplemented with 8 μg/mL polybrene (Sigma) and cells were transduced with the lentivirus‐packaged sgRNA library at a multiplicity of infection (MOI) of 0.3, for a coverage of approximately 300 cells/sgRNA. Transduction was stopped after 24 h by medium change. Cell selection started 38 h after transduction by the addition of puromycin (Gibco) at 0.3 μg/mL in the medium. Medium was supplemented with puromycin until the end of the experiment.

After 2 weeks of puromycin selection, cells were seeded at 450 cell/mm^2^ in 10 transwells (75 mm Transwell® with 3.0‐μm pore polycarbonate membrane insert in 100‐mm dishes, Corning), for a total of 20 × 10^6^ cells. Medium in the top compartment of the transwells was DMEM/1% FBS and in the bottom compartment DMEM/15% FBS, both with 0.3 μg/mL of puromycin. At this passage stage, 10 × 10^6^ cells were frozen and labeled as “Time 0” sample for subsequent analyses. Medium in transwells was changed after 24 h, and cells collected at 48 h from seeding, after PBS washing, by 0.25% trypsin digestion for 3 min at 37°C only in the top compartment, and an additional scraping in the bottom compartment. Cells from top and bottom compartments were seeded in separate T525 at 150 cell/mm^2^ and cultured for 48 h. Then, 8 × 10^6^ cells were frozen and labeled as “Top1” and “Bottom1,” respectively, for subsequent analyses, and 8 × 10^6^ cells were seeded at 450 cell/mm^2^ for a second round of selection. Similar to the procedure above, “Top2” and “Bottom2” samples were collected and frozen, and a third round of selection followed for the collection of samples “Top3” and “Bottom3.” In parallel, transduced cells were cultured in conventional flasks in DMEM/15% FBS, and cells collected at the same time points as the samples from transwells, labeled as “Control1,” “Control2,” and “Control3.”

### 2.5 Genomic DNA extraction and deep sequencing

Genomic DNA was extracted using Quick‐gDNA MidiPrep (Zymo Research) following manufacturer's instructions. Genomic DNA amplification and library preparation for Next Generation Sequencing analysis was performed according to Joung et al.^16^ The library products were sequenced by Genewiz (Suzhou, China) via Illumina HiSeq2000, 2 × 150PE, with 10G coverage and 32.6 M clean reads per sample on average. Illumina binary base call (BCL) files were converted into fastq file format by Illumina bcl2fastq software (v.2.17).

### 2.6 sgRNA deep sequencing data processing

Deep sequencing data from the Crispr‐Cas9 knockout screening were processed for sgRNA representation using custom scripts. Paired reads were merged using Pear^17^ (v.0.9.11), and trimmed using cutadapt^18^ (v.2.8). The trimmed reads were mapped to the human GeCKOv2 library by Bowtie^19^ (v.1.2.3). The sgRNA count matrix was obtained from Bowtie output sam files with a bash script. The percentage of mapped reads was above 80% for all samples.

Data analysis was performed with R software (v.4.2.2). Plots were generated using library ggplot2^20^ v.3.5.0. Gini index was calculated with function *gini.wtd* from library dineq^21^ v.0.1.0, on log2 transformed data. Area‐proportional Venn diagrams were generated using libraries eulerr^22^ v.7.0.0 and ggVennDiagram^23^ v.1.5.2. Raw data were filtered out if sgRNA counts were less than 2^5^ in either Time 0 or Control samples; afterwards, if a gene or miRNA had less than 3 sgRNAs, it was excluded. Each sample was median‐normalized with respect to Time 0 sample, including only the sgRNAs that were within the range mean ± 3 s.d. of the Time 0 distribution. Statistical analysis of sgRNA enrichment was performed using MAGeCK‐VISPR^24^ (v.0.5.6) maximum likelihood estimation (MLE) algorithm, first comparing only Control samples to Time 0 sample, then comparing Bottom samples to Top samples. The obtained beta scores represent the average log‐fold change between the conditions compared for each gene or miRNA. Hierarchical clustering with heat map visualization of the log‐ratio of the sgRNA normalized counts was performed using library pheatmap^25^ v.1.0.12, using Euclidean distance and complete linkage. Heat map visualization of log_2_(sgRNA normalized counts) was performed without clustering, applying the row order obtained from the previous analysis. Genesets used in the functional analysis included essential genes from Hart et al.,^26^ Gene Ontology‐Biological Processes, retrieved using the libraries GO.db^27^ v.3.16.0 and org.Hs.eg.db^28^ v.3.16.0, Reactome pathways (2024), and hierarchy visualization, retrieved through Cytoscape^29^ v.3.10.0 plugin ClueGO^30^ v.2.5.10. Analysis of Reactome pathways in ClueGO included clustering categories according to a score of 0.4 and network visualization.

## 3 RESULTS AND DISCUSSION

### 3.1 Preliminary tests on the transwell functional assay

**FIGURE 1:**
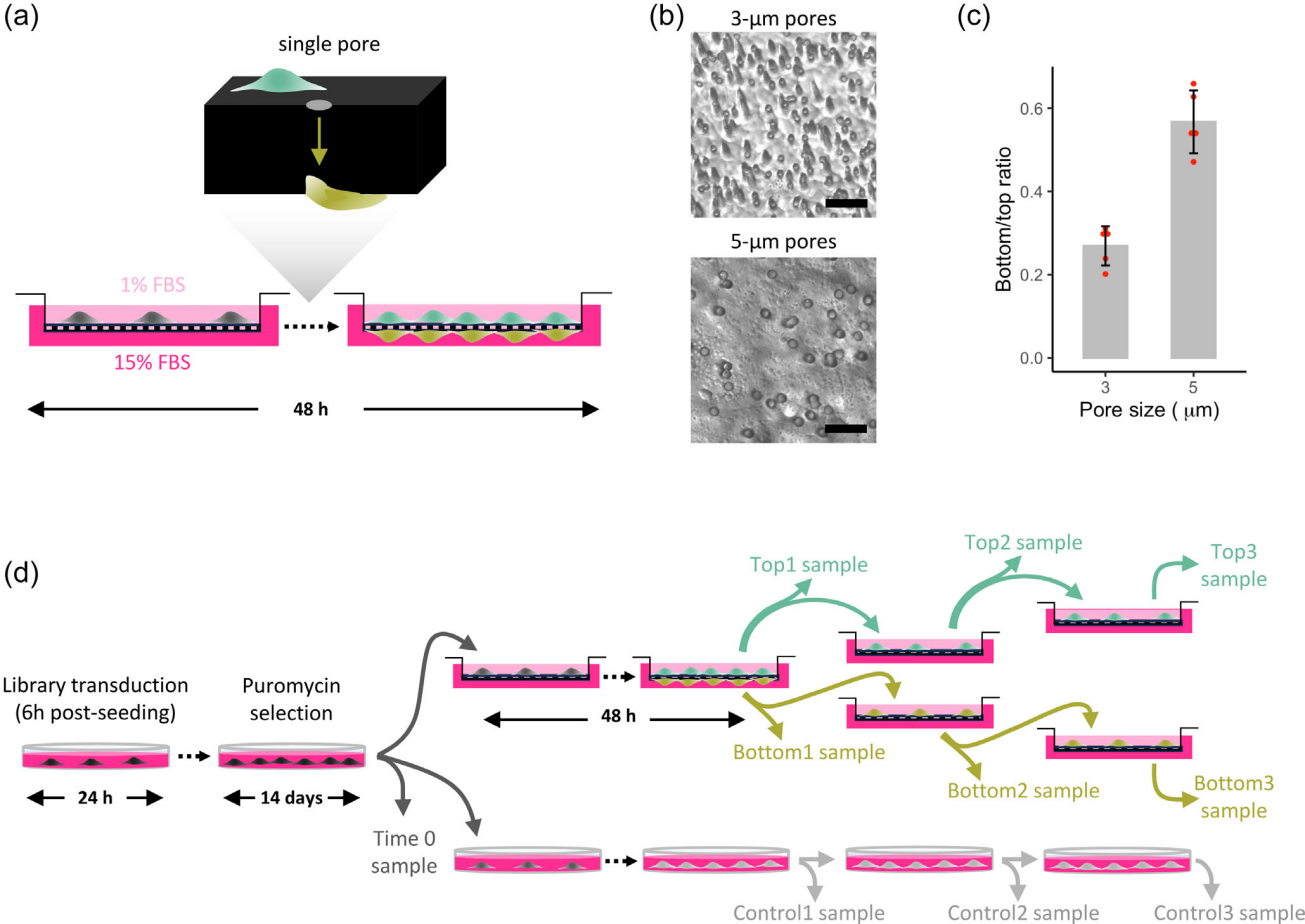
Experimental strategy. (a) Schematic representation of fibroblast migration under the selective pressure of a biochemical gradient, given by a different FBS concentration in the two compartments of the transwell device. (b) Optical microscopy images of the pore size distribution of the transwell membranes with 3‐μm (above) and 5‐μm (below) pore sizes. Scale bar, 20 μm. (c) Ratio of cell density at the bottom face of the membrane to the top face after 48 h of culture in the transwell device with pore sizes shown in (b), under the experimental conditions described in (a). Error bar, mean ± s.d. (*n* = 5). Difference was significant (two‐tailed *t*‐test, *p*‐value = 7 × 10^−5^). (d) Schematic outline of the experimental strategy for the CRISPR‐Cas9 knockout screening. Human fibroblasts were seeded at 200 cell/mm^2^ and transduced, after 6 h, with the GeCKO v.2 lentiviral library. After 24 h, transduction was stopped by medium change and, after a further 38 h, puromycin selection was started for 2 weeks. Then, cells were split to start the selection in 3‐μm pore transwell and, in parallel, expanded within standard dishes. Three rounds of selection were performed.

The transwell migration assay is a commonly used test to study the migratory response of cells in response to a biochemical concentration gradient. In our study, we induced cell migration by using two different concentrations of fetal bovine serum at either side of the transwell membrane (Figure 1a). Human fibroblasts were seeded at 1% serum concentration on the top of the transwell membrane and induced to migrate toward the 15% concentration at the bottom face of the membrane during a 48‐h incubation period. Initially, we tested two membrane pore sizes, 3 and 5 μm (Figure 1b), and found that reducing the pore size correlated with a lower ratio of cells between bottom and top compartments, indicative of a higher selective pressure (Figure 1c). Given these results, the 3‐μm pore condition was considered adequately selective for performing a screening based on this functional assay.

### 3.2 Design of the CRISPR‐Cas9 knockout screen

Human fibroblasts were transduced with the Human CRISPR Knockout Pooled Library (GeCKO v2 library),^15^ which has a lentiviral backbone containing both the *Streptococcus pyogenes* Cas9 nuclease and the sgRNA scaffold and includes puromycin as an antibiotic selection marker. The library spans the whole genome with 3 sgRNAs per 19,051 genes and 4 sgRNAs per 1864 miRNAs. Cells were transduced at a multiplicity of infection of 0.3 and with a coverage of ~300 cells/sgRNA to statistically guarantee at most one virus per cell and a sufficient representation of each sgRNA in the cell population. Afterwards, cells were expanded for 14 days under puromycin selection conditions before starting the migration selective pressure in tranwells (Figure 1d). This expansion aimed at excluding from the cell population all sgRNAs that would be removed even in the absence of a migratory selective pressure, thus sgRNAs targeting genes or miRNAs essential for cellular viability and proliferation under standard culture conditions. This expansion step should restrict the subsequent selection to genes or miRNAs related to the process of directed cell migration under a biochemical gradient and not affecting major cellular functions.

Human fibroblast selection in transwells was performed similarly to the preliminary experiment above, using 3‐μm pore transwell devices. Three rounds of selection were performed, collecting separately the cells attached to the top and the bottom of the membrane. At each round, cells collected from the top compartment were seeded again in the top compartment of new devices, and cells collected from the bottom compartment were seeded at the top of the membrane of other new devices. Between each round of transwell selection, there was a 48‐h expansion in conventional flasks to ensure a sufficient number of cells for collection and re‐seeding. Figure 1d shows the nomenclature of the samples collected, which also includes control samples obtained by expanding fibroblasts in standard culture dishes. At the end of the experiment, the genomic DNA from each sample was sequenced and analyzed to obtain a count matrix for each sgRNA in each sample.

### 3.3 Quality control of CRISPR‐Cas9 knockout screening results

**FIGURE 2:**
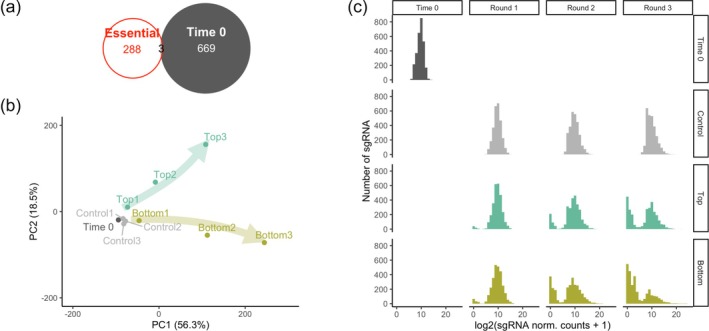
Quality control of filtered normalized data from CRISPR‐Cas9 knockout screening. (a) Venn diagram between core essential genes, as identified by Hart et al.,^26^ and filtered data from Time 0 sample. (b) Principal component analysis (PCA) of sgRNA filtered and normalized counts transformed in log scale. (c) Histogram of the sgRNA count distribution of filtered and normalized data.

The first quality control was performed on the plasmid library, amplified before lentiviral packaging, to verify any potential bias that may affect screening results. Only 4 sgRNAs were lost in the amplification procedure, and the sgRNA distribution had a narrow distribution (Figure S1a). Then, raw data of all samples were analyzed to quantify unevenness of sgRNA distribution and number of missing sgRNAs (Figure S1b,c). Gini index is a measure of sgRNA diversity in culture; the higher the value, the lower the diversity. Gini index has values close to zero for a perfectly even distribution (same number of counts for each sgRNA). Thus, it is expected that Gini index increases while sgRNAs are progressively selected by culture conditions. The Gini index for the plasmid library was as low as 0.074 (Figure S1b), confirming the narrow distribution shown in Figure S1a. Gini index strongly increased in the Time 0 sample (0.141), due to the high number of sgRNAs that were eliminated during the first 14 days of expansion pre‐screening (Figure S1c). We hypothesized that these eliminated sgRNAs are targeting essential genes or miRNAs for fibroblast culture in our experimental conditions. We asked whether these null sgRNAs in the Time 0 sample included those targeting genes recognized to be essential in multiple other cell lines according to a previous study.^26^ Figure 2a shows that all but 3 of these known essential genes were excluded in our Time 0 sample.

We filtered the data according to stringent criteria, excluding sgRNAs that did not have a sufficiently high count^31^ in Time 0 and control samples and genes and miRNAs not targeted by at least 3 sgRNAs. The threshold above^31^ was chosen according to Figure 2c where, in samples with bimodal distribution, this value represents the approximate right end of the negative peak. The sgRNA drop‐out also involved genes and miRNAs that probably affect cell proliferation and fitness more indirectly. In Control samples, we still observed an sgRNA drop‐out, even in the absence of the migratory selective pressure. These sgRNAs were thus filtered out. Principal component analysis (PCA) results showed that Control samples cluster quite closely to the Time 0 sample, without an evident temporal trend (Figure 2b). On the other hand, two diverging temporal trajectories were obtained for Top and Bottom samples, demonstrating the ability of the selection process to progressively highlight differences between the two cell subpopulations. The histograms of the sgRNAs in the filtered data in each sample showed that along the migratory selective process there is a progressive loss of sgRNAs, particularly in the Bottom samples (Figure 2c).

### 3.4 Hit analysis confirms known migration‐related genes

**FIGURE 3:**
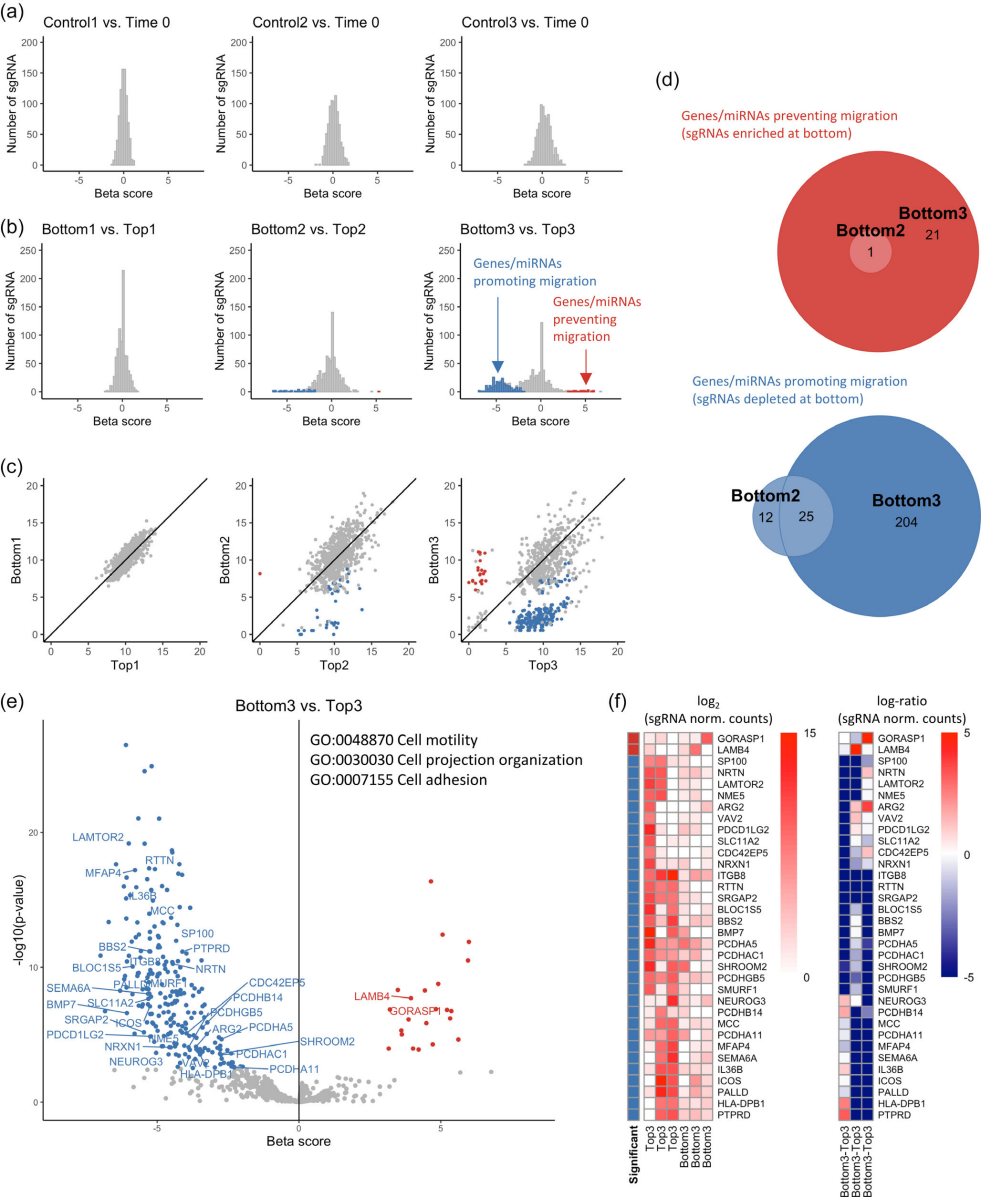
Hit analysis from CRISPR‐Cas9 knockout screening. (a) Histograms of beta scores (equivalent to a mean log‐fold change between the indicated conditions) obtained from MAGeCK‐VISPR algorithm, representing an average log‐fold change for each gene/miRNA in the indicated Control sample with respect to Time 0 sample. No significant differences are detected in each pair comparison. Wald‐FDR <0.01. (b) Histogram of beta scores obtained from MAGeCK‐VISPR algorithm, representing an average log‐fold change for each gene/miRNA in the indicated Bottom sample with respect to the corresponding Top sample. Histograms of the significant differences in each pair comparison are highlighted in blue (depleted at Bottom) and red (enriched at Bottom). Wald‐FDR <0.01. (c) Log mean normalized counts of sgRNAs for each gene/miRNA. Comparison between Bottom and Top samples in each round of the selection. Genes/miRNA identified as significantly different between the two conditions are color‐coded as in (b). (d) Venn diagram of the overlap of significantly different genes/miRNAs identified in the different rounds of selection. (e) Volcano plot of the results from the last round of screening, Bottom3 vs. Top3. Significant genes/miRNAs are highlighted with the same color code as in (c) and labeled if belonging to the genes in the three Gene Ontology‐Biological Processes categories shown. (f) Hierarchical clustering with heat map visualization of migration‐related genes identified in (e). Normalized counts (left) and log‐fold change (right) of each sgRNA targeting the specific gene.

We identified significant hits, genes, and miRNAs whose sgRNAs are enriched or depleted, based on the MAGeCK‐VISPR maximum likelihood estimation algorithm. As mentioned above, after data filtering, each gene and miRNA was targeted by at least 3 sgRNAs to have higher statistical significance. First, we compared the three Control samples with the Time 0 sample to exclude from the following migration analysis genes and miRNAs whose sgRNAs were enriched or depleted under conventional culture conditions. However, due to the initial data filtering, most of the sgRNA selection in normal culture occurred in the first 14 days after transduction, and no additional hit was identified in these comparisons (Figure 3a).

Then, we compared Bottom samples with Top samples at each round of the selection process. Comparing these pairs of samples, instead of comparing each sample with respect to Time 0, reduced potential confounding effects, for example, due to the drop‐out of sgRNAs for migration‐unrelated phenomena and proliferation biases over time. Significant hits were identified from the second round and increased in number in the third round (Figure 3b,c), with the third round results including most of the significant features (genes or miRNAs) from the previous one (Figure 3d). Features whose sgRNAs were depleted in the Bottom compared to the Top samples represent those features that, once deleted, hinder cell migration, and thus will be called promoting migration. On the contrary, features whose sgRNAs were enriched in the Bottom samples are preventing migration because, once deleted, the cell migration propensity is increased. The full list of significantly enriched or depleted hits between Bottom 3 and Top 3 samples is presented in Datasets S1 and S2.

We asked whether known genes involved in migration could be identified in the screening. In particular, we searched for genes belonging to the Gene Ontology–Biological Processes categories “cell motility,” “cell projection organization,” and “cell adhesion.” The last two categories are well known to be related to the process of cell migration because the initial response of a cell to a migration‐promoting concentration gradient is to polarize and extend protrusions that assemble new adhesion points, while at the rear of the cell focal adhesions disassemble.^2^, ^3^ Migration‐related genes that are significant hits are highlighted in Figure 3e,f and listed in Table 1.

**TABLE 1:** List of the significant genes identified in the screening belonging to the indicated gene ontology–biological processes categories.

Gene ontology	Gene ontology	Significant genes
ID	Category name	Bottom3 vs. Top3^a^
GO:0048870 (BP)	Cell motility	BBS2, BMP7, ITGB8, LAMB4, LAMTOR2, MCC, NRTN, PALLD, SEMA6A, SHROOM2, SP100, SRGAP2, VAV2
GO:0030030 (BP)	Cell projection organization	BBS2, BLOC1S5, BMP7, CDC42EP, NEUROG3, NME5, NRTN, NRXN1, PALLD, PTPRD, RTTN, SEMA6A, SLC11A, SMURF1, SRGAP2, SYNE1, VAV2
GO:0007155 (BP)	Cell adhesion	ARG2, BMP7, HLA‐DPB1, ICOS, IL36B, ITGB8, LAMB4, MFAP4, NRXN1, PALLD, PCDHA11, PCDHA5, PCDHAC1, PCDHB14, PCDHGB5, PDCD1LG2, PTPRD, SEMA6A, SRGAP2

^a^ Genes highlighted in Figure 3e,f.

Notably, most genes in these categories, such as all actin genes, were already removed from the screening at Time 0 because of their essential function in cellular survival and proliferation. Among the most significant migration‐promoting genes, the p14‐MP1 complex (LAMTOR2/3) is a known regulator of focal adhesion remodeling, and its absence has been reported to reduce the migration speed^32^; RTTN plays a role in cilia structure and function^33^; MFAP4 is crucial for type I collagen, elastin, and tropoelastin binding^34^; IL36B, formerly known as IL‐1F8, modulates inflammation and fibrosis^35^; MCC and SMURF1 are implicated in migration through the WNT pathway,^36^, ^37^ while CDC42EP5 and VAV2 are involved in Rac‐driven migration.^31^, ^38^, ^39^ Two identified significant genes preventing migration are LAMB4 and GORASP1. LAMB4 belongs to the laminin family, constituents of the extracellular matrix; however, it is still poorly characterized, and it is currently not known to take part in any laminin heterotrimers.^40^, ^41^ The only study on its relation to migration reported that LAMB4 silencing in head and neck squamous cell carcinoma promotes migration, in agreement with what we found in fibroblasts.^42^ GORASP1, also known as GRASP65, is a Golgi structural protein critical in establishing polarity in migrating cells once regulated by phosphorylation.^43^

### 3.5 Hit analysis identifies new targets in migration

**FIGURE 4:**
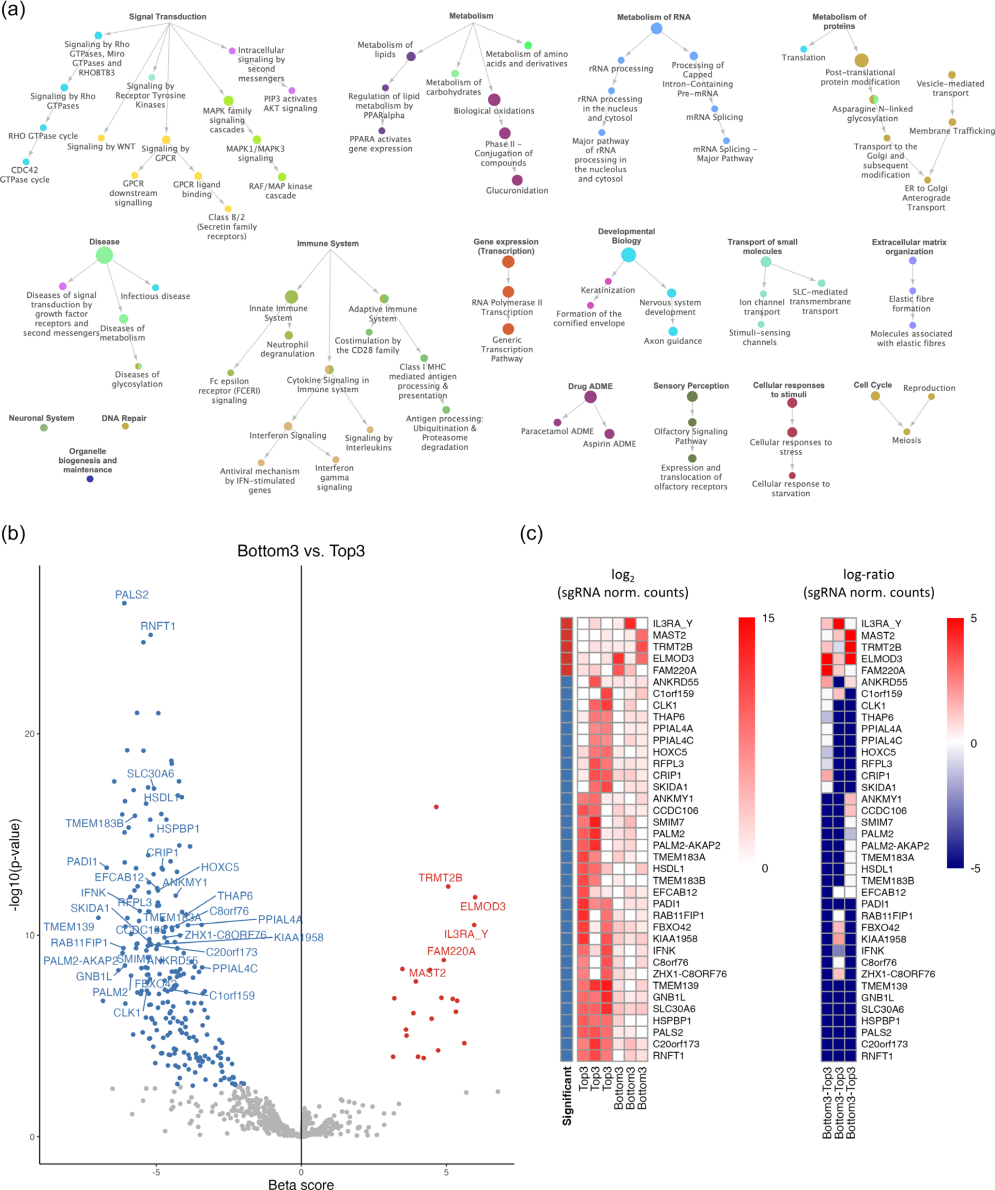
Functional analysis of hits from CRISPR‐Cas9 knockout screening. (a) Identification of Reactome categories with more than 3 significant genes identified from the screening. Node size is proportional to the number of hits in that category. Node color identifies significant clusters (‐score = 0.4). (b) Volcano plot of the results from the last round of screening, Bottom3 vs. Top3. Significant genes/miRNAs are highlighted with the same color code as in (Figure 3d) and labeled if not assigned to any Reactome category in (a), not included in migratory GO categories in Figure 3e, and have a Wald‐FDR < 10^−6^. (c) Hierarchical clustering with heat map visualization of genes labeled in (b). Normalized counts (left) and log‐fold change (right) of each sgRNA targeting the specific gene.

We then analyzed genes not associated with the migratory genesets above. In the CRISPR‐Cas9 knockout screening, every counted sgRNA took action in a single cell where it silenced the targeted feature, gene, or miRNA, at a specific genomic locus. Thus, features with significantly enriched or depleted sgRNAs individually triggered the cell response. This is different from, for example, transcriptomic data where a number of genes collectively describe cell behavior. However, given that biological processes typically include some regulatory redundancy, we asked whether there are multiple hits that could be ascribed to the same biological functions. We selected all Reactome pathways that contained at least three genes that were found significant from the screening. Reactome categories were then visualized according to Reactome hierarchy in Figure 4a.

We found a number of metabolic categories that can be at least partially explained by the biochemical gradient used in the screening, where top cells were exposed to a lower serum concentration compared to bottom cells. These categories are related to the trigger of the migration process in this context. We found Rho GTPase, WNT, and MAPK signaling, together with the extracellular matrix (ECM) organization, which are already known processes related to migration and cancer metastasis.^44^ We also found membrane trafficking, independently of Rho GTPase signaling. Moreover, we identified genes belonging to the transport of small molecules, like ions, which may suggest a role of mechanisms that control cell volume, facilitating the cell squeezing through constrictions, as previously reported for SLC12A6 (a.k.a. KCC3).^45^ We also identified a number of immune‐related genes. Fibroblast role in immune regulation has been described in inflammation, cancer, and infection, also in the context of fibroblast‐secreted soluble and ECM molecules promoting the migration of immune cells.^46^ The identification in this screening may also show a self‐regulation role on fibroblast migration.

Interestingly, a number of hits belonging to the signaling by G protein‐coupled receptors (GPCRs) were detected. In particular, in the Reactome category “Sensory perception,” non‐pseudogene olfactory receptors (OR10H1, OR10K2, OR1E1, OR2A42, OR52D1) and a gene belonging to the family of taste receptors (TAS2R4) were identified. These GPCRs are better known for their role in olfactory and taste recognition,^47^ however, their expression in other tissues has been described, suggesting sensory functionality in other contexts.^48^ OR1E1, OR2A42, OR52D1, and TAS2R4 have already been listed as ectopically expressed,^49^ and OR10H1 has been suggested as a biomarker in urinary tract cancer.^48^, ^50^ Our results found OR10H1, OR10K2, OR1E1, and OR52D1 to promote migration with high significance (Wald‐FDR < 10^−6^). Interestingly, OR2A42 was found to prevent migration (Wald‐FDR < 10^−4^).

The full list of genes belonging to each of the identified Reactome categories is reported in Dataset S3. Figure 4b,c highlights the remaining most highly significant genes that could not be classified in Reactome categories shown in Figure 4a and in migratory GO categories “cell motility,” “cell projection organization,” and “cell adhesion.” They represent an important resource for further experimental studies. Furthermore, in Figures S2 and S3, the full graphical results of significant genes and miRNAs are presented.

## 4 CONCLUSIONS

The CRISPR screening technological field is moving toward a deeper phenotypic characterization of cells subjected to a screening selective pressure, for example by coupling gene editing with single‐cell sequencing or imaging readouts.^5^ However, these types of screening rely on molecular expression and are still limited in their ability to confirm the involvement of the selected features (genes, miRNAs) in the actual cellular function.

This work describes a new methodology that expands the range of applicability of CRISPR screening to directly assay for genetic determinants of cellular function. We successfully coupled CRISPR‐Cas9 knockout pool screening with the transwell migration functional assay. This methodology integrates the advantage of single‐gene or single‐miRNA genetic modification with the statistical power of a bulk functional screening. While this system simplifies the cellular physiological microenvironment (for instance, by lacking components related to mechanical cell interactions), it serves as a robust assay for isolating and analyzing the specific influence of the soluble microenvironment on cell migration.

The results obtained include a list of genes and miRNAs potentially involved in human fibroblast migration under biochemical gradient that can be further validated. While each knocked‐out feature identified by this type of screening may be individually relevant if validated by low‐throughput experiments, we also highlighted some functional categories that impact cell propensity to migrate with multiple genes. Given that the screening was performed excluding all genes and miRNAs related to essential cellular functionalities, it gave the opportunity to avoid targets that could be related to general functions like cytoskeleton components and cell cycle genes.

Interestingly, the screening highlighted multiple genes in the GPCR family of proteins, in particular ectopically expressed olfactory and taste receptors, which are currently recognized to be upregulated in several cancers.^48^ This result points out the relevance of biochemical sensing upstream of the migratory process itself in response to a biochemical gradient. These GPCRs have high therapeutic potential, given their variety and the specificity of their biochemical recognition.^48^ Targeting the identified GPCRs represents a promising strategy for translating our findings into potential therapeutic avenues, particularly in the context of pathological migration and tumor metastasis. On the other hand, the specific receptors involved in the migratory process are presumably very context‐dependent, depending on the biochemical gradient occurring in the extracellular environment.

We envision that the presented screening methodology, which can be applied also with more targeted CRISPR‐Cas9 knockout sub‐libraries, could support hit identification in therapeutically relevant contexts. With more knowledge accumulating about olfactory receptor ligands,^16^ screening this GPCR class for involvement in migration can give a mechanistic understanding of the biochemical triggers of the process and open new therapeutical perspectives.

## AUTHOR CONTRIBUTIONS

Antonio Mazzei was involved in data curation and formal analysis. Sebastian Martewicz was involved in methodology and experimental investigation. Ramin Amiri supported formal analysis and results visualization. Meihua Cui performed CRISPR‐Cas9 library expansion and was involved in resource collection. Sebastian Martewicz, Camilla Luni, and Nicola Elvassore were involved in conceptualization. All authors were involved in review and editing of the manuscript. Nicola Elvassore and Camilla Luni were involved in funding acquisition. Camilla Luni was involved in original draft writing and project supervision.

## CONFLICT OF INTEREST STATEMENT

The authors have no conflict of interest to declare.

## Supporting information


**Figure S1.** Quality control of CRISPR‐Cas9 library and raw data of all samples. (a) Histogram of the distribution of sgRNAs in the plasmid library. (b) Gini index, as a measure of sgRNA unevenness within the cell population, of the indicated samples (raw data). (c) Number of sgRNAs with zero counts, i.e. not detected, in the indicated samples (raw data).
**Figure S2.** Significant genes identified in the screening. Hierarchical clustering with heat map visualization of all significant genes identified in the screening. Normalized counts (a) and log‐fold change (b) of each sgRNA targeting the specific gene. On the left each row has been annotated with the main Ractome functional categories shown in Figure 4a. Wald‐FDR <0.01.
**Figure S3.** Significant miRNAs identified in the screening. Hierarchical clustering with heat map visualization of all significant miRNAs identified in the screening. Normalized counts (a) and log‐fold change (b) of each sgRNA targeting the specific miRNA. Some miRNAs were targeted by 4 sgRNAs, a gray box indicates that for that miRNA only 3 sgRNAs were present. Wald‐FDR <0.01.

**Dataset S1.** MAGeCK‐VISPR results of all genes and miRNAs, performed from the filtered normalized data.

**Dataset S2.** List of all genes and miRNAs annotated for significance with a Wald‐FDR <0.01.

**Dataset S3.** List of clusters and genes for Reactome categories shown in Figure 4a.

## Data Availability

The data that support the findings of this study are openly available in Gene Expression Omnibus (GEO) at https://www.ncbi.nlm.nih.gov/geo/, reference number GSE266226.
