# Supplementary material for: Functional CRISPR‐Cas9 knockout screening of the genetic determinants of human fibroblast migration propensity
Source: Biotechnol Prog. 2025 Oct 2;42(1):e70076. doi: 10.1002/btpr.70076 (PMC12908101; doi:10.1002/btpr.70076)
Supplement: Supplementary file 1 — Figure S1. Quality control of CRISPR‐Cas9 library and raw data of all samples. (a) Histogram of the distribution of sgRNAs in the plasmid library. (b) Gini index, as a measure of sgRNA unevenness within the cell population, of the indicated samples (raw data). (c) Number of sgRNAs with zero counts, i.e. not detected, in the indicated samples (raw data). Figure S2. Significant genes identified in the screening. Hierarchical clustering with heat map visualization of all significant genes identified in the screening. Normalized counts (a) and log‐fold change (b) of each sgRNA targeting the specific gene. On the left each row has been annotated with the main Ractome functional categories shown in Figure 4a. Wald‐FDR <0.01. Figure S3. Significant miRNAs identified in the screening. Hierarchical clustering with heat map visualization of all significant miRNAs identified in the screening. Normalized counts (a) and log‐fold change (b) of each sgRNA targeting the specific miRNA. Some miRNAs were targeted by 4 sgRNAs, a gray box indicates that for that miRNA only 3 sgRNAs were present. Wald‐FDR <0.01. [file BTPR-42-e70076-s001.pdf]

Supporting Information

Functional CRISPR-Cas9 knockout screening of the genetic determinants of human fibroblast migration propensity

Antonio Mazzei[1,#], Sebastian Martewicz[2,#], Ramin Amiri[1], Meihua Cui[2], Nicola Elvassore[3,4], Camilla Luni[1,*]

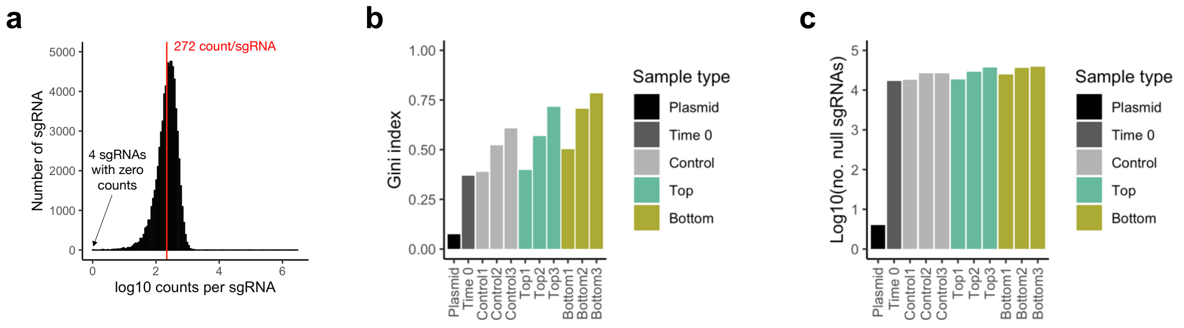

Figure S1. Quality control of CRISPR-Cas9 library and raw data of all samples. (a) Histogram of the distribution of sgRNAs in the plasmid library. (b) Gini index, as a measure of sgRNA unevenness within the cell population, of the indicated samples (raw data). (c) Number of sgRNAs with zero counts, i.e. not detected, in the indicated samples (raw data).

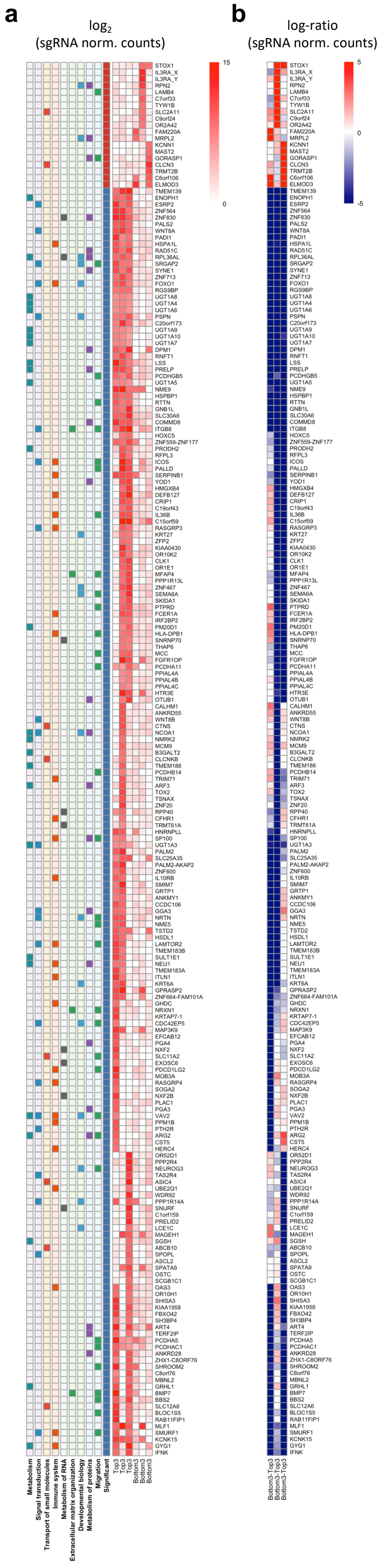

a
log2 (sgRNA norm. counts)
15
0
Metabolism
Signal transduction
Transport of small molecules
Immune system
Metabolism of RNA
Extracellular matrix organization
Developmental biology
Metabolism of proteins
Migration
Significant
Top3
Top3
Top3
Bottom3
Bottom3
Bottom3
b
log-ratio (sgRNA norm. counts)
5
0
-5
Bottom3-Top3
Bottom3-Top3
Bottom3-Top3

Figure S2. Significant genes identified in the screening. Hierarchical clustering with heat map visualization of all significant genes identified in the screening. Normalized counts (a) and log-fold change (b) of each sgRNA targeting the specific gene. On the left each row has been annotated with the main Ractome functional categories shown in Figure 4a. Wald-FDR < 0.01.

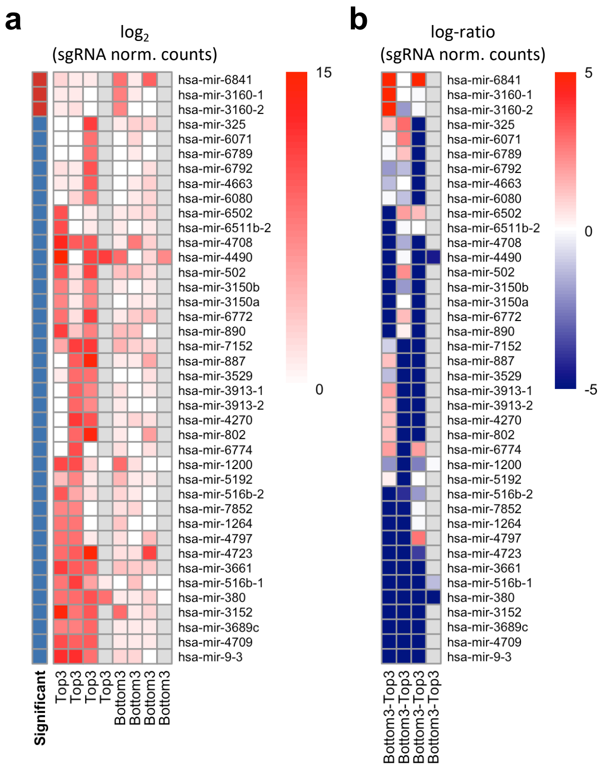

Figure S3. Significant miRNAs identified in the screening. Hierarchical clustering with heat map visualization of all significant miRNAs identified in the screening. Normalized counts (a) and log-fold change (b) of each sgRNA targeting the specific miRNA. Some miRNAs were targeted by 4 sgRNAs, a gray box indicates that for that miRNA only 3 sgRNAs were present. Wald-FDR < 0.01.

Supplementary dataset 1. MAGeCK-VISPR results of all genes and miRNAs, performed from the filtered normalized data.

Supplementary dataset 2. List of all genes and miRNAs annotated for significance with a Wald-FDR<0.01.

Supplementary dataset 3. List of clusters and genes for Reactome categories shown in Figure 4a.
